# Metallo-β-lactamase-producing *Pseudomonas aeruginosa*: epidemiology, diagnostic challenges and therapeutic strategies in the era of MDR/XDR resistance

**DOI:** 10.3389/fmicb.2026.1924442

**Published:** 2026-09-04

**Authors:** Alessandra Imeneo, Alessandro Capone, Valentina Antonelli, Valentina Perri, Ivano Petriccione, Alessandra Lodi, Carla Fontana, Stefania Cicalini

**Affiliations:** 1 Systemic and Immune Depression-Associated Infections Unit, National Institute for Infectious Diseases “Lazzaro Spallanzani”, IRCCS, Rome, Italy; 2 Microbiology and Biobank Unit, National Institute for Infectious Diseases “Lazzaro Spallanzani”, IRCCS, Rome, Italy

**Keywords:** carbapenem resistance, cefiderocol, difficult to treat, metallo beta lactamase (MBL), *Pseudomonas aeruginosa*

## Abstract

**Background:**

The management of multidrug-resistant (MDR) *Pseudomonas aeruginosa* has evolved from an antibiotic-centric approach to a mechanism-driven paradigm. Among resistance determinants, metallo-β-lactamases (MBLs) represent a critical challenge due to their ability to hydrolyse most β-lactams and their increasing global dissemination within high-risk clones.

**Methods:**

This narrative review aims to summarize the epidemiology, diagnostic approaches and therapeutic strategies for infections caused by MBL-producing *P. aeruginosa*, emphasizing a mechanism-based clinical framework. A PubMed-MEDLINE search was conducted up to 1 June 2026 using the terms “*Pseudomonas aeruginosa*”, “*carbapenem resistance*”, and “*metallo-*β*-lactamase*”. Relevant studies were selected based on the authors' assessment of methodological quality and clinical relevance.

**Results:**

*P. aeruginosa* exhibits multifactorial resistance involving intrinsic mechanisms (efflux pumps, AmpC overexpression, porin loss), adaptive responses and acquired carbapenemases. MBLs, especially VIM, IMP, and emerging NDM variants, are increasingly associated with carbapenem resistance and epidemic high-risk clones (e.g., ST235, ST111). Global epidemiology shows marked geographic variability in prevalence and molecular types. Diagnostic workup remains challenging due to the coexistence of enzymatic and non-enzymatic mechanisms, requiring integrated use of phenotypic antibiotic susceptibility testing (AST), carbapenemase detection assays, molecular diagnostics, and, in selected cases, whole-genome sequencing (WGS). Among treatment, cefiderocol represents the most evidence-supported option for MBL-producing strains. Aztreonam-based combinations offer an alternative treatment option, provided susceptibility is confirmed by synergy testing. Emerging antimicrobials (e.g., taniborbactam-, xeruborbactam-, and zidebactam-containing regimens) and novel strategies such as bacteriophage therapy are regarded as promising alternatives. Evidence for combination therapy remains limited and should be individualized.

**Conclusions:**

MBL-producing *P. aeruginosa* requires an integrated, mechanism-based diagnostic and therapeutic strategy incorporating local epidemiology, rapid diagnostics and optimized antimicrobial selection. Ongoing development of novel agents and precision microbiology tools is expected to further refine clinical management.

## 1 Introduction

The management of multidrug-resistant (MDR) Gram-negative infections has undergone a profound transformation over the past decade. For many years, therapeutic decision-making was largely antibiotic-centered, relying primarily on the *in vitro* susceptibility profile of the infecting isolate. In this traditional paradigm, clinicians selected antimicrobial agents based on the lowest minimum inhibitory concentration (MIC) or the broadest apparent spectrum of activity, often without explicit consideration of the molecular mechanisms of resistance. Although this approach was appropriate when therapeutic options were limited and resistance mechanisms were relatively predictable, it has become increasingly inadequate in the era of complex multidrug resistance.

Among Gram-negative pathogens, *Pseudomonas aeruginosa* exemplifies this challenge. Unlike species in which resistance is frequently driven by a single acquired determinant, *P. aeruginosa* exhibits a highly dynamic and multifactorial resistome. It harbors a broad array of intrinsic and acquired resistance mechanisms, alongside adaptive resistance such as biofilm formation and the emergence of multidrug-tolerant cells, which contribute to infection persistence and relapse (Pang et al., 2019). As a result, isolates with similar phenotypic susceptibility profiles may differ substantially in their underlying resistance mechanisms, risk of therapeutic failure, and propensity to further develop resistance during treatment.

Intrinsic resistance mechanisms include low outer membrane permeability, overexpression of efflux pumps and the production of antibiotic-inactivating enzymes, such as AmpC β-lactamase. In addition, *P. aeruginosa* readily acquires resistance through the emergence of spontaneous chromosomal mutations and horizontal gene transfer mediated by integrons and other mobile genetic elements. The global spread of high-risk clones further contributes to the dissemination of MDR and epidemic spread, reflecting their enhanced capacity to acquire and maintain resistance determinants. Hypermutation, defined as an increased spontaneous mutation rate, is frequently observed in *P. aeruginosa* and facilitates rapid adaptation to fluctuating environments, including the airways of patients with cystic fibrosis (Pang et al., 2019; Oliver et al., 2025; Zhai et al., 2025; Botelho et al., 2019; Oliver et al., 2000).

Several definitions of antimicrobial resistance in *P. aeruginosa* have been proposed. According to the European Society of Clinical Microbiology and Infectious Diseases (ESCMID) guidelines, MDR strains are non-susceptible to at least one agent in three or more antimicrobial categories; *extensively drug-resistant* (XDR) strains are non-susceptible to at least one agent in all but two or fewer antimicrobial categories whereas *pandrug-resistant* (PDR) strains are non-susceptible to all agents in all antimicrobial categories. More recently, the Infectious Diseases Society of America (IDSA) introduced the concept of *difficult-to-treat resistance* (DTR) to better capture clinically relevant resistance phenotypes. DTR *P. aeruginosa* is defined as resistance to all first-line antimicrobial agents, including carbapenems, piperacillin-tazobactam, ceftazidime, cefepime, ciprofloxacin and levofloxacin. These isolates represent a major therapeutic challenge, as treatment often relies on newly developed antimicrobial agents or older drugs associated with increased toxicity and adverse effects (Magiorakos et al., 2012; Paul et al., 2022; Livermore et al., 2020; Tamma et al., 2024).

Carbapenem-resistant *P. aeruginosa* (CRPA) is of particular concern, as these isolates frequently exhibit co-resistance to multiple antimicrobial classes, resulting in limited therapeutic options and an increased risk of treatment failure. The combination of intrinsic resistance mechanisms, acquisition of transferable resistance determinants, and the ability to disseminate in healthcare settings explains why *P. aeruginosa* has remained classified among the high-priority pathogens in both the previous and the updated World Health Organization (WHO) Bacterial Priority Pathogens Lists, highlighting the urgent need to develop of new antimicrobials (Tacconelli et al., 2018).

Although several β-lactam/β-lactamase inhibitor (BL-BLI) combinations have recently expanded therapeutic options for MDR Gram-negative infections, their clinical use has been accompanied by increasing selective pressure and the emergence of resistance mechanisms that may compromise long-term efficacy.

Among these mechanisms, carbapenemase production, especially by metallo-β-lactamases (MBLs), has been increasingly reported in CRPA, although it remains less prevalent than in *Enterobacterales*. MBL-producing *P. aeruginosa* poses a major therapeutic challenge, due to reduced susceptibility to both traditional and newer β-lactam agents. In this context, current IDSA guidelines recommend cefiderocol, a novel siderophore cephalosporin, as a preferred treatment option for infections caused by MBL-producing *P. aeruginosa* (Tamma et al., 2024). However, identification of carbapenemase-producing CRPA remains challenging because standardized EUCAST screening recommendations are lacking and routine carbapenemase testing is not universally implemented across clinical microbiology laboratories (Ellem et al., 2026).

This narrative review summarizes the epidemiology, diagnostic strategies and therapeutic management of infections caused by MBL-producing *P. aeruginosa* within a mechanism-based therapeutic framework. We discuss how resistance mechanisms influence antimicrobial activity, the biological rationale underlying therapeutic strategies, and the available clinical evidence. Finally, we propose a practical algorithm for individualized treatment of infections caused by MBL-producing *P. aeruginosa*.

### 1.1 Methods

The scope of this narrative review was defined to include the following topics: the epidemiology of MBL-producing *P. aeruginosa*, with particular emphasis on regional variability; diagnostic strategies and clinical management; currently available therapeutic options and future perspectives. The relevant literature was subsequently identified through a MEDLINE/PubMed literature search conducted up to 1 June 2026. PubMed was selected as the primary source because of its extensive coverage of high-quality biomedical literature and its suitability for the scope of the present review. The search strategy included the terms “*Pseudomonas*”, “*Pseudomonas aeruginosa*”, and “*P. aeruginosa*”, combined with “carbapenem resistance”, “MBL”, and “metallo-β-lactamase”. No restrictions were applied regarding study design, whereas articles not published in English were excluded. No additional databases (e.g., Embase or Scopus) were searched. Relevant guidelines, systematic reviews, and meta-analyses were also considered. Although no time limits were imposed, particular attention was given to studies published within the last 10 years. Eligible studies included original research articles, observational studies, clinical studies, surveillance studies, and relevant reviews addressing the epidemiology, diagnosis, treatment, or clinical management of MBL-producing *P. aeruginosa*. Articles were selected through discussion among the authors based on their relevance to the objectives of the review and the quality of the available evidence, assessed using a hierarchical approach based on study design. When conflicting evidence was identified, priority was given to higher-quality studies and to findings that were consistently supported across multiple independent studies. In addition, backward and forward citation tracking (snowballing) was also performed to identify further relevant studies not captured by the initial database search.

## 2 Metallo-β-lactamase-mediated resistance in *Pseudomonas aeruginosa*

Carbapenems are pivotal therapeutic agents for the treatment of severe Gram-negative bacterial infections. Consequently, CRPA poses a major clinical threat due to the limited treatment options available and the risk of nosocomial transmission. Several studies have associated CRPA infections with poorer clinical outcomes, largely because of the increased likelihood of inappropriate empirical antimicrobial therapy, the high burden of comorbidities, and the prolonged hospitalization (Horcajada et al., 2019).

**Figure 1:**
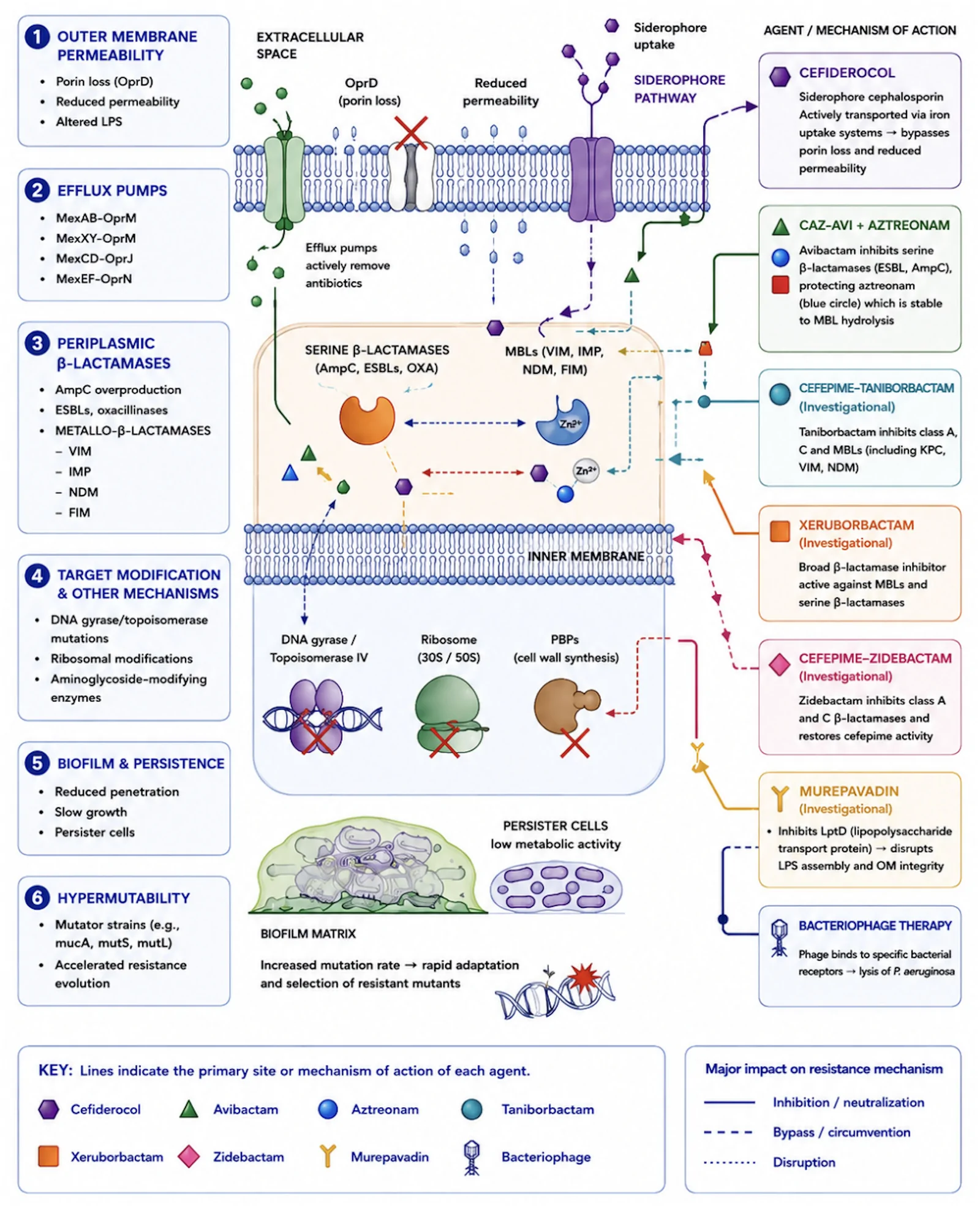
Major resistance mechanisms and therapeutic targets in carbapenem-resistant *Pseudomonas aeruginosa*. AmpC, class C β-lactamase; CAZ-AVI, ceftazidime-avibactam; ESBL, extended-spectrum β-lactamase; FIM, florence imipenemase; IMP, imipenemase; KPC, *Klebsiella pneumoniae* carbapenemase; LPS, lipopolysaccharide; LptD, lipopolysaccharide transport protein D; MBL, metallo-β-lactamase; Mex, multidrug efflux system; NDM, New Delhi metallo-β-lactamase; OM, outer membrane; OprD, outer membrane porin D; OXA, oxacillinase; PBP, penicillin-binding protein; VIM, Verona integron-encoded metallo-β-lactamase. Schematic overview of the major resistance mechanisms in carbapenem-resistant *Pseudomonas aeruginosa* and the mechanisms of action of current and emerging antimicrobial agents, highlighting strategies to overcome reduced permeability, efflux pumps, β-lactamase production, target modification, and biofilm-associated persistence. Solid lines indicate direct inhibition or neutralization, dashed lines indicate bypass or circumvention of resistance mechanisms, and dotted lines indicate disruption of bacterial targets or cellular processes. Figure design was assisted by ChatGPT (OpenAI).

In *Enterobacterales*, carbapenem resistance is primarily mediated by the production of different types of carbapenemases. Conversely, carbapenem resistance in *P. aeruginosa* may arise through multiple distinct mechanisms (Figure 1). Moreover, these mechanisms frequently coexist within the same isolate, complicating both diagnosis and treatment while increasing the likelihood of cross-resistance to multiple antimicrobial classes (Falcone et al., 2024).

One important mechanism is the overexpression of efflux pumps, especially MexAB-OprM, which may reduce susceptibility to several β-lactams and non-β-lactam agents, with the notable exception of imipenem. Another major mechanism involves chromosomal AmpC hyperproduction combined with loss or downregulation of OprD porin, which substantially contributes to β-lactam resistance (Ellem et al., 2026; Livermore, 2002; Lister et al., 2009; Poole, 2004; González-Pinto et al., 2024). Beyond these well-characterized mechanisms, additional adaptive processes further increase the complexity of antimicrobial resistance in *P. aeruginosa*. Mutations affecting antimicrobial targets—including DNA gyrase, topoisomerase IV, ribosomal structures, and aminoglycoside-modifying pathways—may reduce susceptibility to non-β-lactam agents. Furthermore, biofilm formation and persistence phenotypes promote antimicrobial tolerance, whereas hypermutator strains carrying defects in DNA repair pathways may accelerate the emergence and accumulation of resistance determinants (Figure 1).

Finally, acquisition of carbapenemase genes through horizontal gene transfer represents an additional resistance mechanism. This mechanism is particularly concerning because carbapenemase-producing CRPA has been increasingly reported worldwide and carries a substantial risk of dissemination within healthcare settings (Reyes et al., 2023; Ahmadi et al., 2025; Malkoçoglu et al., 2017; Li et al., 2026). Among carbapenemases, MBLs are the predominant enzymes, followed by Ambler Class A enzymes (such as KPC or carbapenem-hydrolyzing GES variants) and selected Class D oxacillinases (mainly OXA-10-like variants, and, much more rarely, carbapenem-hydrolyzing enzymes such as OXA-198), although their distribution varies considerably across geographic regions (Ahmadi et al., 2025; Mancini et al., 2025; Findlay et al., 2024; Tenover et al., 2022). Among these, MBLs are particularly important because they hydrolyse most β-lactams, including carbapenems, and are not inhibited by currently available β-lactamase inhibitors such as avibactam, relebactam or vaborbactam (Cornaglia et al., 2011; Oliver et al., 2024).

Numerous MBL variants have been identified in *P. aeruginosa*. Among these, Verona integron-encoded metallo-β-lactamase (VIM) is the most widespread, followed by imipenemase-type metallo-β-lactamases (IMP) (Yoon and Jeong, 2021). Several additional MBL variants have been reported with regional heterogeneity, including New Delhi MBL (NDM) and several geographically restricted enzymes (e.g., SPM, GIM, SIM, AIM, DIM, FIM, HMB, KHM, TMB, and related lineages), which show marked regional heterogeneity (Jabalameli et al., 2018; Hong et al., 2015; Yoon and Jeong, 2021; Pollini et al., 2013).

A major epidemiological concern is the dissemination of blaMBL genes within epidemic high-risk clones of *P. aeruginosa*. These lineages, notably ST235, ST111, and ST233, are key drivers of global dissemination in healthcare settings. Their success is largely attributable to the ability to acquire and maintain mobile resistance determinants, including β-lactamases such as extended spectrum β-lactamases (ESBLs) and carbapenemases, thereby enhancing their MDR phenotype (Horcajada et al., 2026; Del Barrio-Tofiño et al., 2017). Among high-risk clones, ST235 is the most widely distributed and clinically relevant lineage, while ST111 and ST233 are frequently associated with blaVIM-2. In contrast, ST277 is strongly linked to SPM-type MBL production (Zhai et al., 2025; Oliver et al., 2024; Molina-Mora et al., 2020). Importantly, the global distribution of these clones is dynamic and shaped by local antimicrobial pressures, infection control practices, healthcare networks and international patient movement. The interaction between successful clonal lineages and mobile genetic elements contributes to the emergence of a heterogeneous and globally disseminated population of MBL-producing *P. aeruginosa*, complicating both epidemiological tracking and therapeutic decision-making.

Although carbapenemase production is not the predominant mechanism of carbapenem resistance in CRPA, its emergence has major therapeutic implications (Hidalgo-Tenorio et al., 2024). MBLs hydrolyze virtually all β-lactams, with the exception of monobactams. Consequently, MBL-producing *P. aeruginosa* isolates are generally resistant to both traditional antipseudomonal β-lactams (ceftazidime, cefepime, piperacillin-tazobactam) and the newer BL-BLI combination (ceftolozane-tazobactam, imipenem-relebactam and ceftazidime-avibactam). Reduced susceptibility to fluoroquinolones and aminoglycosides is frequently observed and resistance determinants targeting these antimicrobial classes are often co-located with blaMBL genes on plasmids, integrons or other mobile genetic elements (Tenover et al., 2022).

## 3 Epidemiology of carbapenem resistance and MBL production in *Pseudomonas aeruginosa*

Over the past three decades, carbapenem resistance in *Pseudomonas aeruginosa* has evolved from being predominantly driven by chromosomal mechanisms (such as OprD loss, AmpC overexpression, and efflux pump upregulation) to the increasing global dissemination of acquired MBLs. Unlike *Enterobacterales*, MBL-producing *P. aeruginosa* typically harbors multiple additional resistance mechanisms, reflecting the organism's remarkable genomic plasticity and resulting in highly MDR phenotypes.

Most epidemiological studies on CRPA have been geographically restricted or have lacked molecular characterization of the isolates. In addition, because carbapenemase screening in CRPA is not routinely performed in most diagnostic laboratories, particularly for isolates with carbapenem MICs close to clinical breakpoints, the reported prevalence is likely underestimated (Reyes et al., 2023; Mancini et al., 2025; Horcajada et al., 2019; Li et al., 2026).

The transferable β-lactamases, including ESBL and carbapenemases, especially MBLs, are increasingly reported worldwide, although with substantial geographic variability. Reported prevalence varies substantially according to the study population, geographic setting and molecular epidemiology (Horcajada et al., 2019; Juan et al., 2013). Marked geographic variability in carbapenemase prevalence has been reported among CRPA, ranging from approximately 2% in the United States to 69% in Central and South America, to 57% in Australia and Singapore, to 30% in the Middle East and to 32% in China, as reported in a large multicentric prospective cohort study (Reyes et al., 2023). Furthermore, a recent meta-analysis warned about the increasing prevalence of resistance to meropenem in CRPA from the year 1997 to 2023, reporting pooled resistance rates of approximately 23% for meropenem and 33% for imipenem (Ahmadi et al., 2025).

These data highlight the marked geographic variability in the distribution of carbapenem resistance mechanisms among CRPA isolates. Given this variability, the present review provides a brief overview of global epidemiology before focusing on the European context, with particular emphasis on Italy, where MBL-producing *P. aeruginosa* represents a major clinical concern.

### 3.1 Global epidemiology of MBL-producing *P. aeruginosa*

In a multicenter study including CRPA isolates recovered from bloodstream, respiratory, urine, and wound specimens collected across 44 hospitals in 10 countries, MBL production was identified in more than 20% of isolates (Reyes et al., 2023). Similarly, a recent global genomic analysis based on *P. aeruginosa* genomes available in the National Center for Biotechnology Information (NCBI) database reported an overall MBL prevalence of 12%, with blaVIM being the most frequently identified gene (51%), followed by blaIMP (24%) and blaNDM (23%) (Zhai et al., 2025). Notably, considerably higher prevalence of blaMBL genes have been reported in Iran (77%) and Pakistan (76%) (Jabalameli et al., 2018; Ali et al., 2021).

The dissemination of MBL-producing *P. aeruginosa* has evolved from an initial dominance of IMP enzymes in East Asia and VIM enzymes in Europe and Latin America to a more complex global scenario, now significantly reshaped by the emergence and spread of NDM. This distribution continues to shift, with the occasional appearance of rare enzymes such as FIM, reflecting ongoing horizontal gene transfer and the pathogen's high adaptability, sometimes leading to regional replacement of established MBL types. Surveillance studies on clinical MBL-producing *P. aeruginosa* isolates indicate that VIM enzymes are globally distributed and have been reported across all six continents. IMP enzymes represent the second most prevalent MBL variants worldwide. In contrast, NDM-producing isolates appear to be predominantly reported in Asia, Europe and Africa (Yoon and Jeong, 2021). Other MBL variants, including SPM, GIM, FIM, and AIM, have been sporadically reported and display a more restricted geographic distribution, being mainly associated with Brazil, Germany, Italy, and Australia, respectively (Botelho et al., 2019).

Overall, available evidence indicates that the global epidemiology of MBL-producing *P. aeruginosa* remains highly dynamic and is driven by the expansion of successful high-risk clones together with the horizontal dissemination of mobile resistance determinants.

**Figure 2:**
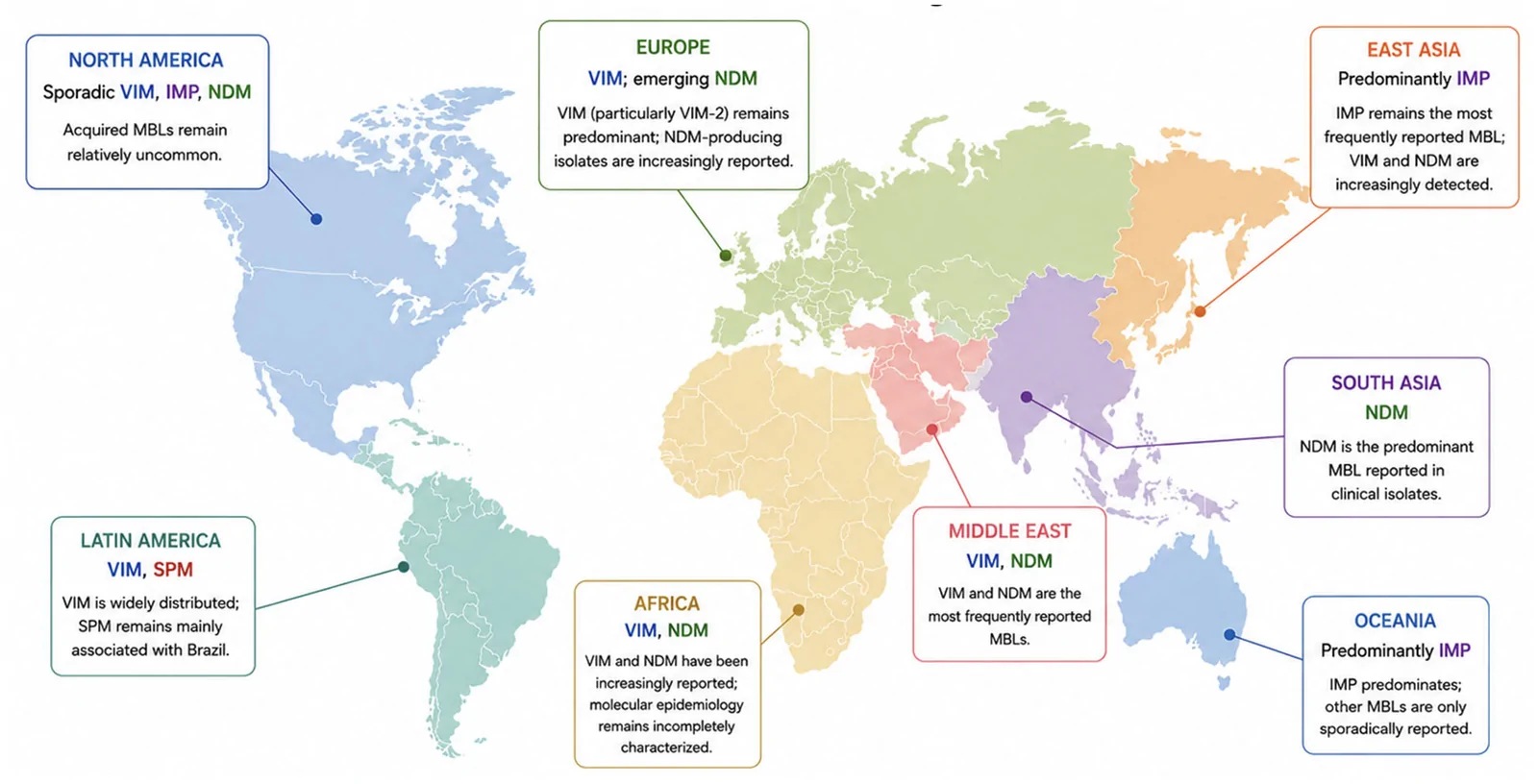
Global distribution of the predominant metallo-β-lactamases (MBLs) reported in *Pseudomonas aeruginosa*. IMP, imipenemase; MBL, metallo-β-lactamase; NDM, New Delhi metallo-β-lactamase; SPM, São Paulo metallo-β-lactamase; VIM, Verona integron-encoded metallo-β-lactamase. The figure summarizes the predominant MBL families reported across different geographic regions based on the studies included in this review. Distribution patterns may vary according to local molecular epidemiology, healthcare setting and surveillance period. Figure design was assisted by ChatGPT (OpenAI).

The global distribution of the predominant MBLs reported in *P. aeruginosa* is summarized in Figure 2.

### 3.2 Current epidemiology in Europe

**Figure 3:**
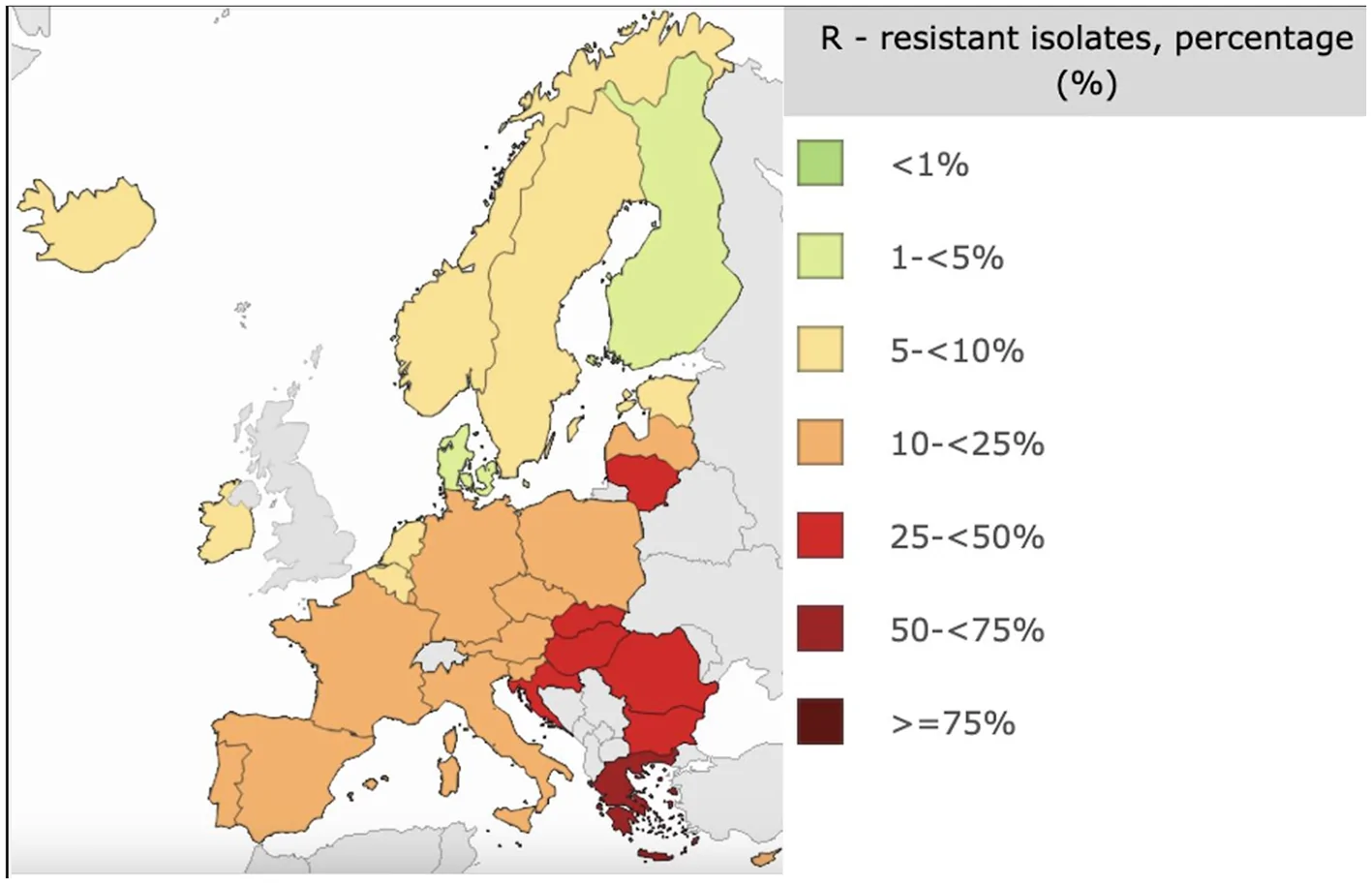
Distribution of carbapenem-resistance *Pseudomonas aeruginosa* isolates (percentage) in Europe in 2024. Adapted from the surveillance atlas of infectious diseases of the European Centre for Disease Prevention and Control (ECDC). Available at https://www.ecdc.europa.eu/en/surveillance-atlas-infectious-diseases.

Surveillance data from the European Antimicrobial Resistance Surveillance Network (EARS-Net)^1^ indicate that carbapenem resistance remains widespread across Europe, with the highest rates reported in Southern and Eastern European countries (Figure 3). Recent surveillance reports also suggest an increasing burden of invasive CRPA infections across the European region. Considerable heterogeneity persists among European countries, with carbapenem resistance rates ranging from less than 5% in countries such as Iceland and Norway to 50% or higher in Greece, Romania, Bulgaria and Cyprus.

Nevertheless, recent surveillance data also suggest stabilization, or even a decline, in carbapenem resistance among bloodstream isolates in several European countries between 2020 and 2024. These findings should be interpreted cautiously, as antimicrobial resistance continues to represent a major public health concern across Europe. Molecular epidemiological studies indicate that this burden is largely driven by the dissemination of high-risk clones and the spread of MBL, particularly VIM-type enzymes.

European surveillance systems mainly report phenotypic rates of carbapenem resistance in *P. aeruginosa*, without routinely characterizing the underlying resistance mechanisms. Consequently, current data on MBL-producing *P. aeruginosa* in Europe are predominantly obtained from national or multicenter molecular studies. While blaVIM-2 remains the predominant MBL determinant circulating in Europe and is associated with a limited number of successful high-risk clones, blaNDM-1 showed a broader geographical distribution and greater genetic diversity (Fortunato et al., 2023). These findings are supported by a Swiss molecular surveillance study, in which VIM enzymes accounted for 54% of all MBL-producing *P. aeruginosa* isolates, followed by NDM and IMP enzymes (28% and 15%, respectively). However, recent reports from Greece have documented the rapid emergence and dissemination of NDM-producing clones, resulting in a shift in local epidemiology and replacement of the previously predominant VIM-producing strains (Protonotariou et al., 2024).

### 3.3 Current epidemiology in Italy

The Annual Epidemiological Report of the European Centre for Disease Prevention and Control (ECDC)^2^ reported an increase in both the number of participating Italian laboratories and the number of reported *P. aeruginosa* isolates between 2020 and 2024. However, the estimated incidence of CRPA bloodstream infections remained overall stable during the same period.

According to Italian National Institute of Health (ISS),^3^ the proportion of invasive *P. aeruginosa* isolates resistant to the major antimicrobial classes has shown a gradual decline between 2015 and 2024. In particular, carbapenem resistance increased during 2020–2021 (16%−17%), remained relatively stable during 2022–2023 (approximately 16%), and subsequently declined to 14% in 2024. Nevertheless, substantial regional heterogeneity persists. The lowest incidence of CRPA has been reported in Friuli-Venezia-Giulia (7%), followed by Emilia-Romagna and Trento (8% and 9%, respectively), whereas the highest rates were observed in Basilicata (29%), followed by Molise, Umbria, Lazio and Calabria (ranging from 19% to 21%).

In parallel with carbapenem resistance, reduced susceptibility to recently introduced BL-BLI combinations is emerging as an additional concern. In Italy, CRPA isolates collected in 2024 exhibited resistance rates of 24% to ceftazidime–avibactam, 23% to ceftolozane–tazobactam and 42% to imipenem–relebactam. These values appear higher than those reported in previous Italian studies, suggesting a possible worsening trend that warrants continued surveillance (Imeneo et al., 2025; Valzano et al., 2024; Morroni et al., 2022).

National surveillance data specifically addressing MBL-producing *P. aeruginosa* in Italy are currently lacking. Consequently, published prevalence varies substantially according to geographical region and clinical setting. A recent large multicenter study conducted in Italian intensive care units reported a CRPA incidence of 1.74 per 1,000 patient-days, with CRPA accounting for 27% of all *P. aeruginosa* infections. Among the limited subgroup of isolates subjected to genomic characterization, MBL-producing *P. aeruginosa*, predominantly carrying VIM-enzymes, were detected among CRPA isolates (De Pascale et al., 2025).

## 4 Diagnostic challenges in carbapenem-resistant *Pseudomonas aeruginosa*: from resistance detection to mechanism-informed clinical interpretation

CRPA remains one of the most difficult antimicrobial resistance phenotypes to interpret in routine clinical microbiology. The diagnostic challenge extends beyond simply detecting resistance to imipenem or meropenem. The key clinical question is whether carbapenem resistance is mediated by an acquired carbapenemase, chromosomal non-enzymatic mechanisms or a combination of both. This distinction has important implications for infection control, epidemiological surveillance, and antimicrobial therapy. Unlike carbapenem-resistant *Enterobacterales*, in which carbapenemase production is often the primary diagnostic target, carbapenem resistance in *P. aeruginosa* is frequently multifactorial (Lister et al., 2009; Oliver et al., 2024). Resistance reflects the interplay of multiple mechanisms, including β-lactamase production, reduced outer membrane permeability due to porin loss, efflux pump overexpression, target modifications, biofilm formation, and regulatory mutations driving hypermutability and intrinsic resistance pathways (Figure 1).

Furthermore, in cases where *P. aeruginosa* is repeatedly isolated from the same patient, guidelines recommend performing repeat antibiotic susceptibility testing (AST) to monitor the emergence of resistance (Tamma et al., 2024). This approach is justified by evidence demonstrating that resistance may arise even against antimicrobial agents that have never been directly administered to the patient, underscoring the dynamic and unpredictable nature of resistance evolution in *P. aeruginosa* (Imeneo et al., 2025; Jørgensen et al., 2013; Wardell et al., 2019).

The main diagnostic questions in CRPA, together with the available diagnostic methods, their analytical principles, turnaround times, key limitations, and clinical implications, are summarized in Table 1. This framework highlights that no single diagnostic approach is sufficient to fully characterize CRPA resistance and that complementary methods are required to integrate phenotypic, enzymatic, and genomic information.

**Table 1:** Diagnostic questions, available methods and key limitations in carbapenem-resistant *Pseudomonas aeruginosa*.

Diagnostic questions	Methods	Analytical principle	Turnaround time	Key limitations	Clinical and therapeutic implications
Is the isolate carbapenem resistant?	Standard AST (disk diffusion, automated systems)	Standard phenotypic growth measurement in the presence of carbapenems	16–24 h	Does not define the underlying resistance mechanism	Essential first step; guides initial empirical therapy but not mechanism-based decisions
	Automated rapid AST (rAST)	Real-time kinetic monitoring: tracks single-cell bacterial growth under antibiotic pressure via microfluidics or automated imaging	2–6 h	High mechanistic resolution requires secondary confirmatory testing	Bypasses simple “enzyme-presence” tests by providing full, quantitative MICs for BL and non-BL; critical for early targeted therapy
Is resistance mediated by a carbapenemase?	Carba NP test	Colorimetric pH assay: measures reaction medium acidification driven by carbapenemase-mediated imipenem hydrolysis	< 2 h	Lower sensitivity for weak carbapenemase producers or certain OXA/MBL variants; no MIC data	Supports rapid infection control and early therapeutic orientation (e.g., immediate suspicion of MBLs)
	Traditional CIM (mCIM/sCIM)	Biological disk inactivation assesses imipenem hydrolysis by the test strain using a susceptible *E. coli* reporter lawn	16–24 h	Long incubation delay; may be affected by bacterial permeability and expression levels	Gold standard phenotypic confirmation officially adopted by CLSI, but delayed results impact timely stewardship
	Rapid CIM (rCDM/rCIM)	Accelerated biological inactivation: uses thin agar plates (3 mm) and dense indicator inocula (3.0 McFarland) to accelerate visual zone inhibition reading	5–6 h	May miss very weak producers (e.g., low-expression VIM-4); entirely unsuitable for slow-acting oxacillinases (e.g., OXA-23)	Drastically reduces the diagnostic window while maintaining high CRPA performance (97.4% sensitivity, 100% specificity for KPC, VIM, IMP)
Which specific carbapenemase is present?	Molecular and lateral flow (immuno-chromatographic assays, PCR panels)	Targeted amplification of resistance genes (e.g. *bla*_VIM_, *bla*_KPC_) or antigen detection via specific antibodies	< 1–2 h	Detects only predefined targets; misses rare/novel variants; provides no data on chromosomal mechanisms	Critical for immediate MBL detection (tailoring cefiderocol or aztreonam combo) and epidemiological surveillance
Could resistance be non-enzymatic?	AST-based algorithms and comparative BL-BLI panels	Indirect inference: comparing differential susceptibility patterns across multiple newer BL-BLI combinations	16–24 h (or 2–6h via rAST)	Difficult to isolate and quantify the precise mathematical contribution of each individual mechanism	Explains PCR-negative CRPA; prevents misclassification; prioritizes confirmatory tests; crucial since non-enzymatic resistance is multifactorial
Can molecular data reliably predict phenotype?	WGS and targeted sequencing	High-throughput sequencing for comprehensive detection of resistance genes, mutations (*oprD, cirA*), and clonal lineages	Days (retrospective)	Gene presence does not reliably predict MICs due to complex, synergistic interactions of expression levels and efflux	Invaluable for global surveillance, outbreak investigation, and retrospective interpretation of complex MDR isolates

AST, antimicrobial susceptibility testing; BL, β-Lactam; BLI, β-Lactamase inhibitor; BL-BLI, β-lactam/β-lactamase inhibitor; Carba NP, carbapenemase Nordmann–Poirel; CIM, Carbapenem inactivation method; CLSI, Clinical and Laboratory Standards Institute; CRPA, Carbapenem-resistant *Pseudomonas aeruginosa*; IMP, imipenemase; KPC, *Klebsiella pneumoniae* carbapenemase; MBL, metallo-β-Lactamase; mCIM, modified carbapenem inactivation method; MDR, multidrug-resistant; MIC, minimum inhibitory concentration; OXA, oxacillinase; PCR, polymerase chain reaction; rCDM, rapid carbapenemase detection method; rCIM, rapid carbapenem inactivation method; sCIM, simplified carbapenem inactivation method; VIM, Verona integron-encoded metallo-β-lactamase; WGS, whole genome sequencing.

The table reports the diagnostic approaches for carbapenem-resistant *Pseudomonas aeruginosa* and highlights the complementary roles of phenotypic, molecular and genomic methods within a stepwise diagnostic workflow.

### 4.1 Multifactorial resistance and the limits of phenotypic interpretation

The first major diagnostic challenge in CRPA is that carbapenem resistance does not necessarily reflect a single underlying mechanism. Consequently, similar susceptibility patterns may arise from different resistance mechanisms, whereas the same determinant may produce variable phenotypic expression depending on its level of expression and the presence of additional resistance pathways (Lister et al., 2009; Poole, 2004; González-Pinto et al., 2024).

This distinction is not merely academic. Misclassifying a non-carbapenemase-producing CRPA isolate as a suspected carbapenemase producer may lead to unnecessary molecular testing, excessive infection-control measures and inaccurate epidemiological interpretation. Conversely, failure to recognize a true carbapenemase-producing isolate may delay infection-control interventions and affect early therapeutic decisions. The key diagnostic question is not simply whether an isolate is resistant to carbapenem, but rather which mechanism is responsible for the observed phenotype and how this information should guide further investigation and clinical management (Wang et al., 2025; Hassall et al., 2024).

### 4.2 Conventional antibiotic susceptibility testing: indispensable but not mechanistically definitive

Conventional AST remains the cornerstone of CRPA diagnosis because it directly measures bacterial growth under antibiotic exposure and provides MIC values that are essential for guiding antimicrobial therapy. However, as summarized in Table 1, conventional AST does not define the underlying resistance mechanism. This limitation is especially relevant in laboratories that use carbapenem non-susceptibility as the sole trigger for carbapenemase testing, as carbapenem resistance alone has limited specificity for carbapenemase production in *P. aeruginosa* due to the frequent contribution of intrinsic or adaptive mechanisms. Accordingly, several studies have explored AST-based screening algorithms that combine carbapenem results with susceptibility to cephalosporins, aminoglycosides or newer BL-BLI combinations to better identify isolates that warrant confirmatory testing (Vallabhaneni et al., 2021; Gill et al., 2022; Mancini et al., 2025; Ellem et al., 2026).

### 4.3 Phenotypic carbapenemase tests: useful but vulnerable to biological interference

Phenotypic carbapenemase assays detect carbapenem hydrolysis rather than inferring resistance from MIC values. Available methods include inhibitor-based assays (e.g., EDTA or dipicolinic acid for MBLs, and boronic acid derivatives for class A carbapenemases or AmpC-related activity), rapid biochemical assays (including Carba NP and related colorimetric tests), and carbapenem inactivation methods (CIM, mCIM, sCIM).

However, in *P. aeruginosa* and other non-fermenting Gram-negative bacilli, these approaches are susceptible to interference from biological factors such as outer membrane permeability changes (e.g., OprD loss), low-level enzyme expression, or weak hydrolytic activity, leading to false-positive or false-negative results. Consequently, phenotypic carbapenemase tests should be interpreted in conjunction with the full susceptibility profile and cannot replace expanded antimicrobial susceptibility testing (Jing et al., 2019; Tamma and Simner, 2018; Nordmann et al., 2020; Humphries, 2020). The comparative characteristics of these approaches are summarized in Table 1, showing that rapid phenotypic assays provide faster detection but require cautious interpretation in CRPA.

### 4.4 Molecular assays: rapid answers, but only to predefined questions

Molecular assays are particularly useful when the diagnostic question is clearly defined. PCR-based or multiplex platforms can rapidly detect the most clinically relevant carbapenemase genes within a few hours, enabling early infection control measures, outbreak recognition, and the initial guidance for antimicrobial therapy (Boolchandani et al., 2019; Oliver et al., 2024). Nevertheless, these assays detect only the resistance genes included in their panels, while uncommon or locally emerging variants may be missed, as well as chromosomal resistance mechanisms that are not detected. As reported in Table 1, molecular assays provide rapid detection of specific carbapenemase genes but may miss uncharacterized or chromosomal resistance mechanisms. For this reason, a negative molecular result should be interpreted as “*no targeted carbapenemase gene detected*” rather than as “*no clinically relevant resistance mechanism present*” (Lister et al., 2009; Boolchandani et al., 2019; Hassall et al., 2024). Consequently, molecular results in CRPA should be interpreted in conjunction with the phenotypic profile, especially in cases of discordance.

### 4.5 Whole-genome sequencing: high resolution, but not yet a standalone clinical answer

Whole-genome sequencing (WGS) provides the most comprehensive characterization of the resistance background of CRPA. It can identify acquired carbapenemase genes and chromosomal mutations, reconstruct the genetic context of resistance determinants, detect mobile genetic elements and define high-risk clonal lineages associated with hospital outbreaks (Ellington et al., 2017; Didelot et al., 2012; Oliver et al., 2024).

Beyond antimicrobial resistance, WGS characterizes the accessory genome, including virulence-associated traits such as secretion systems, quorum sensing, adhesion, and biofilm formation, which contribute to persistence and chronic infection (Pajaro-Castro et al., 2025; Zhang et al., 2025). It also provides insight into regulatory networks and adaptive mechanisms relevant in healthcare settings, such as disinfectant tolerance. Overall, WGS informs not only the resistome but also the virulome and the adaptive capacity of clinical isolates, helping to explain the success of high-risk clones (Pantanella et al., 2026). This high level of resolution is particularly valuable for epidemiological surveillance and outbreak investigation, as it allows differentiation of clonal spread from independent emergence of resistance mechanisms (Oliver et al., 2024).

However, WGS is not yet a replacement for AST. Genotypic findings do not always correlate with MICs, and the combined phenotypic effect of multiple mechanisms remains difficult to predict, particularly for newer agents influenced by β-lactamases, permeability, efflux activity, and additional pathways such as iron transport systems. Furthermore, cost, infrastructure, turnaround time and bioinformatic expertise still limit routine implementation in many clinical laboratories (Goldberg et al., 2015; Ellington et al., 2017; Hassall et al., 2024).

### 4.6 Phenotypic algorithms: a pragmatic but locally dependent solution

Because no single test can fully resolve the diagnostic complexity of CRPA, several groups have proposed pragmatic, AST-based algorithms to identify isolates with a higher probability of carbapenemase production. These strategies are attractive because they rely on susceptibility results already available in routine laboratories and can reduce unnecessary confirmatory testing.

Vallabhaneni et al. (2021) showed that carbapenem resistance alone has limited specificity for predicting carbapenemase genes, whereas the inclusion of additional susceptibility markers, such as ceftazidime and/or cefepime and/or ceftolozane/tazobactam, improves diagnostic discrimination. Gill et al. (2022) further validated a phenotypic screening strategy based on carbapenem resistance combined with ceftazidime and cefepime non-susceptibility in a multicentre surveillance context, although some carbapenemase-positive isolates, especially GES producers, may be missed. More recently, Mancini et al. developed a mechanism-oriented algorithm that integrates susceptibility to novel BL-BLI combinations and confirmatory immunochromatographic testing. This approach is relevant because it explicitly addresses the difficulty of differentiating MBL/ESBL-producing CRPA from AmpC-hyperproducing isolates using conventional β-lactams alone (Mancini et al., 2025). Finally, Ellem et al. (2026) proposed a simplified screening workflow based on meropenem, ceftazidime and tobramycin, reporting high sensitivity and specificity in their setting; however, their findings also underline the importance of local carbapenemase epidemiology, including the predominance of GES enzymes in their isolate collection.

The principal limitation of these algorithms is their limited generalizability, as performance is strongly influenced by local carbapenemase epidemiology, the prevalence of non-enzymatic resistance mechanisms, the antimicrobial panel adopted and the interpretive criteria applied. Accordingly, phenotypic algorithms should be regarded as screening tools rather than definitive methods for mechanistic classification.

### 4.7 Toward an integrated diagnostic workflow

Accurate diagnosis of CRPA is essential because treatment decisions depend on both the resistance phenotype and the underlying resistance mechanisms, whose identification guides therapy but does not replace phenotypic susceptibility testing (González-Pinto et al., 2024; Hilbert et al., 2023; Tamma et al., 2024). The major challenge is balancing speed, accuracy, mechanistic insight and clinical actionability.

Current EUCAST^4^ and CLSI^5^ recommendations endorse a stepwise approach for CRPA diagnosis, with routine AST serving as the initial screening tool and phenotypic, immunochromatographic, or molecular methods used for confirmatory carbapenemase testing. However, specific guidance for carbapenemase detection in *P. aeruginosa* remains more limited than for *Enterobacterales*, reflecting the complexity of resistance mechanisms in this species.

Distinguishing screening from confirmation is essential, as carbapenem resistance in *P. aeruginosa* is frequently driven by non-enzymatic mechanisms with different implications for infection control and therapy.

A rational workflow should begin with high-quality AST, which provides the basis for both therapeutic decisions and targeted carbapenemase testing. When carbapenem resistance is detected, the susceptibility profile should be interpreted to assess the likelihood of carbapenemase production and to guide confirmatory testing. In routine practice, this corresponds to a stepwise approach in which initial AST directs subsequent diagnostic investigations. Rapid molecular and immunochromatographic assays can identify clinically relevant carbapenemases, particularly MBLs (Oliver et al., 2024). Phenotypic carbapenemase assays may indicate hydrolytic activity, but in *P. aeruginosa* they require cautious interpretation, due to the possible contribution of non-enzymatic resistance mechanisms (Tamma and Simner, 2018). In cases of discordant results, unusual resistance to novel agents or outbreak investigations, WGS or targeted sequencing can provide additional resolution (Ellington et al., 2017; Oliver et al., 2024).

Overall, accurate diagnosis of CRPA relies on an integrated approach, combining phenotypic AST, targeted carbapenemase detection, genomic methods and local epidemiology, with careful integration of results to avoid overinterpretation of any single diagnostic modality. As outlined in Table 1, an integrated workflow combining phenotypic testing with targeted resistance detection and genomic analysis when required balances speed, mechanistic resolution and clinical utility.

## 5 Therapeutic strategies

In critically ill patients with suspected CRPA infection, timely initiation of effective antimicrobial therapy is a major determinant of clinical outcome, as delays in administering an active agent have been associated with increased mortality. However, susceptibility data are rarely available when initial treatment decisions are needed, requiring clinicians to rely on local epidemiology and patient-specific risk factors to guide empirical therapy. Early identification of patients at risk for MBL-producing *P. aeruginosa* infection is essential. Moreover, when a CRPA infection is confirmed, antimicrobial susceptibility testing for the newer β-lactams (ceftolozane-tazobactam, ceftazidime-avibactam, imipenem-cilastatin-relebactam, cefiderocol) is warranted (Tamma et al., 2024). Currently available treatment options for CRPA are summarized in Table 2.

**Table 2:** Currently available antimicrobial options for carbapenem-resistant *Pseudomonas aeruginosa* (CRPA).

Agent	Adult dose (normal renal function)	Activity against MBL-producing *P. aeruginosa*
Cefiderocol	2 g IV q 8 h	Yes
Ceftolozane/tazobactam	Uncomplicated cystitis: 1.5 g q 8 h	No
All other infections: 3 g IV q 8 h	
Imipenem/cilastatin/relebactam	1.25 g IV q 6 h	No
Ceftazidime/avibactam	2.5 g IV q 8 h	No
Aztreonam + ceftazidime/avibactam	Aztreonam 2 g q 8 h + ceftazidime/avibactam 2.5 g q 8 h	Variable
Colistin	Loading-dose 9 MIU, then 4.5 MIU q 12 h	Variable
Amikacin	Once-daily weight-based dosing	Variable
Fosfomycin	4–8 g IV q 6–8 h	Variable

Adapted from the IDSA Guidance on the Treatment of Antimicrobial-Resistant Gram-Negative Infections (2024), ESCMID Guidelines (2022), and the Italian intersociety guidelines (2022) (Tamma et al., 2024; Paul et al., 2022; Tiseo et al., 2022).

### 5.1 Cefiderocol

**Figure 4:**
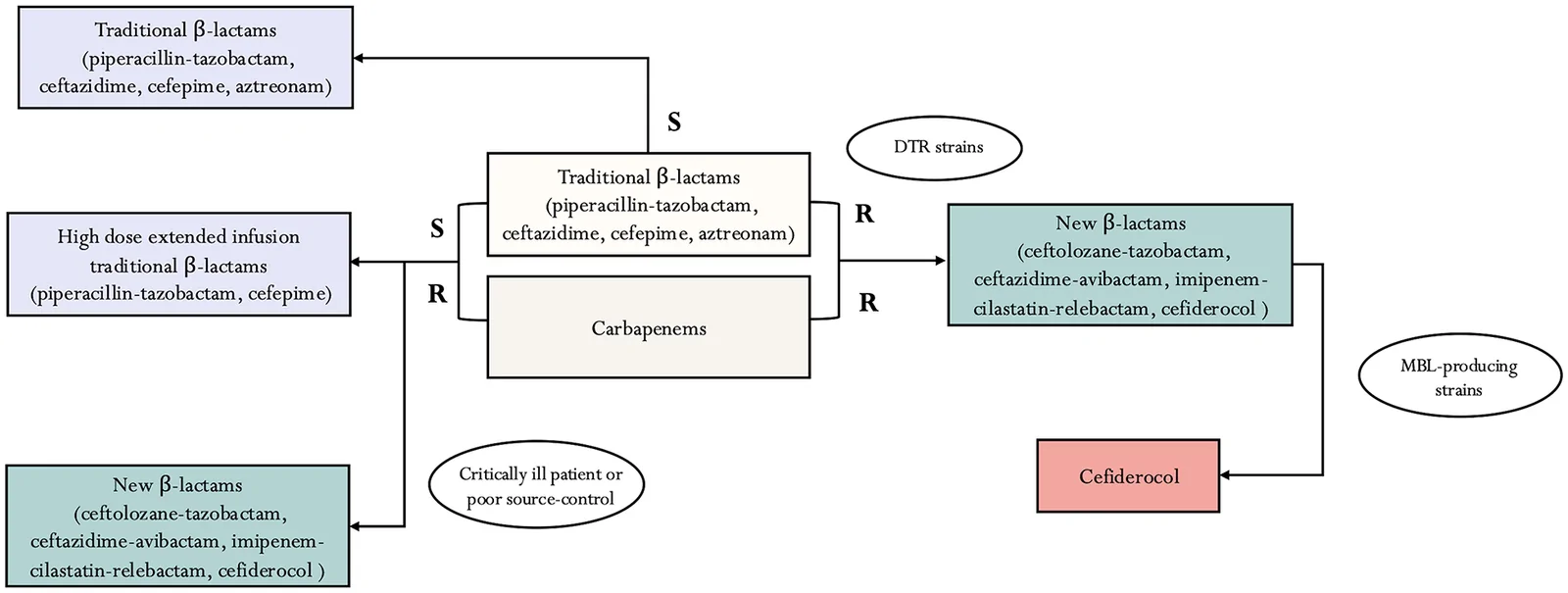
Graphical representation of the Infectious Diseases Society of America (IDSA) guidelines for the treatment of infections due to *Pseudomonas aeruginosa*. DTR, difficult-to-treat resistance; MBL, metallo-β-lactamase; R, resistant isolates; S, susceptible isolates. Figure was prepared based on the IDSA guidelines (Tamma et al., 2024). With the aim to spare both carbapenems and the novel β-lactam/β-lactamase inhibitor (BL-BLI) combinations, guidelines recommend the preferred adoption of the traditional BL-BLI combinations, when susceptible. Conversely, the novel BL-BLI combinations are recommended in DTR *P. aeruginosa* infections or in case of critical patients or when source-control is not feasible. Cefiderocol is the preferred treatment option for MBL-producing *P. aeruginosa*.

According to the IDSA guidelines, traditional BL-BLI combinations should be preferred whenever susceptibility is confirmed, thereby preserving carbapenems and the newer BL-BLI agents. Conversely, novel BL-BLI combinations are recommended for DTR *P. aeruginosa* infections, in critically ill patients, or when adequate source control cannot be achieved. Among the newer agents, cefiderocol is currently considered the preferred treatment for infections caused by MBL-producing *P. aeruginosa*, because of its retained activity against these isolates (Figure 4) (Tamma et al., 2024). Cefiderocol is a siderophore cephalosporin that exploits active iron transport systems to enter bacterial cells and inhibits peptidoglycan synthesis through binding to penicillin-binding protein 3 (PBP3). This unique mechanism allows cefiderocol to retain activity despite porin loss, efflux pump overexpression and carbapenemase production, as consistently demonstrated *in vitro* (Gill et al., 2024; Santerre Henriksen et al., 2024; Malisova et al., 2023; Kimbrough et al., 2025; Wise et al., 2023; McCreary et al., 2021). The drug is commercially available in several countries, including the United States, Japan and many European countries. However, its availability varies worldwide depending on national regulatory approval, reimbursement policies, and hospital formulary adoption.

Resistance remains relatively uncommon in *P. aeruginosa* and is primarily associated with mutations affecting the catalytic region of AmpC, iron acquisition pathways, siderophore receptors and efflux pump overexpression, all of which reduce intracellular drug accumulation. Higher cefiderocol MICs have also been reported among NDM-producing isolates (Gomis-Font et al., 2023; López-Causapé et al., 2023; Karakonstantis et al., 2022; Ito et al., 2017; Russo and Serapide, 2025; Gill et al., 2024; Li et al., 2026). Because susceptibility is influenced by multiple mechanisms, molecular detection alone is insufficient and phenotypic testing remains essential. EUCAST recommends broth microdilution in iron-depleted medium as the reference method, with disk diffusion as a complementary approach, since standard susceptibility methods may not adequately reproduce siderophore-mediated uptake (González-Pinto et al., 2024; Oliver et al., 2024).

Evidence for cefiderocol derives from the GAME CHANGER trial, in which cefiderocol demonstrated non-inferiority but not superiority to standard-of-care therapy with respect to 14-day mortality in patients with Gram-negative bloodstream infections. Interpretation of the findings in patients with MBL-producing *P. aeruginosa* remains limited by the small sample size, precluding species-specific conclusions (Paterson et al., 2026). Further, clinical evidence comes from *post-hoc* analysis of the pivotal CREDIBLE-CR trial and the APEKS clinical development program (Bassetti et al., 2021; Timsit et al., 2022). In CREDIBLE-CR, cefiderocol showed efficacy against a broad range of carbapenem-resistant Gram-negative pathogens, including MBL-producing *P. aeruginosa*, while analyses from the APEKS studies provided additional support for its effectiveness and safety profile. However, the number of enrolled patients with MBL-producing *P. aeruginosa* was limited. Real-world data from the PERSEUS study further strengthens the evidence base (Torre-Cisneros et al., 2025). This large multicentre Spanish cohort included 261 patients treated for severe Gram-negative infections, including 174 *P. aeruginosa* isolates, of which 73 were MBL-producing *P. aeruginosa*. Despite the observational and non-randomized design, cefiderocol achieved a clinical cure rate of 85% (147/174) and a 28-day mortality of 17% (30/174).

Although cefiderocol is currently recommended for the treatment of MBL-producing *P. aeruginosa*, no randomized clinical trial has been specifically designed to evaluate its efficacy in this setting. Therefore, current recommendations are based on the integration of *in vitro* susceptibility data, subgroup and *post hoc* analyses, and observational real-world studies.

### 5.2 Combination therapy

The role of combination therapy remains controversial. Current guidelines generally discourage routine use once an active agent has been identified, due to the limited and inconclusive supporting evidence (Tamma et al., 2024). In the absence of randomized controlled trials, several reports have explored cefiderocol-based combinations with companion agents such as imipenem-relebactam, ceftazidime/avibactam, ampicillin/sulbactam, fosfomycin, aminoglycosides, polymyxins or carbapenems, primarily with the aim of enhancing synergistic activity and reducing the risk of resistance (Granata et al., 2025; Bavaro et al., 2021; Gill et al., 2023; Marino et al., 2022; Tascini et al., 2023). Reported benefits of combination therapy are constrained by substantial selection bias, as it is preferentially used in the most severe and refractory infections, and no randomized trial has yet evaluated mechanism-guided combination therapy vs. optimized monotherapy in MBL-producing *P. aeruginosa*.

Combination therapy may still be considered in selected critically ill patients, particularly in the presence of septic shock, persistent bacteremia, high bacterial burden, inadequate source control, or infection sites with limited antibiotic penetration (Li et al., 2026; Russo and Serapide, 2025). However, available evidence remains limited, and no randomized controlled trials have demonstrated superiority over optimized cefiderocol monotherapy. Accordingly, routine adoption cannot be recommended on the basis of current data. Consistent with this, recent Italian guidelines do not support routine combination therapy for CRPA infections (Tiseo et al., 2022). Nevertheless, the addition of a second active agent—preferably fosfomycin—may be considered on a case-by-case basis in selected severe or complex clinical scenarios.

Combination therapy should be regarded as an individualized, mechanism-based strategy aimed at exploiting complementary pharmacological and microbiological mechanisms, rather than as a universal approach, with its potential benefit depending on the infecting isolate, infection characteristics, and risk of resistance emergence.

### 5.3 Ceftazidime-avibactam plus aztreonam

The combination of aztreonam and avibactam is well-established for the treatment of MBL-producing *Enterobacterales*, as it retains activity in the presence of co-produced MBLs and serine β-lactamases, including ESBL and carbapenemases. However, comparable efficacy has not been demonstrated in *P. aeruginosa*. A systematic review of *in vitro* and clinical studies evaluating aztreonam and avibactam combination reported efficacy rates approaching 80% in MBL-producing *Enterobacterales*, but below 10% in MBL-producing *P. aeruginosa* (Mauri et al., 2021). Further *in vitro* evidence, based on time-kill assays and pharmacokinetic/pharmacodynamic (PK/PD) chemostat models, has compared cefiderocol with ceftazidime-avibactam plus aztreonam in CRPA. Although both strategies showed activity, cefiderocol was the only monotherapy to consistently achieve bactericidal effects across tested isolates (Montero et al., 2025).

Several studies have shown that avibactam provides little or no benefit over aztreonam alone in MBL-producing *P. aeruginosa*, largely due to additional resistance mechanisms that reduce susceptibility. These include overexpression of the MexAB-OprM efflux system, coproduction of AmpC enzymes or other β-lactamases capable of hydrolyzing aztreonam (Testa et al., 2015; Jorth et al., 2017; Pina-Sánchez et al., 2025; Blanco et al., 2025). Moreover, aztreonam monotherapy has been associated with suboptimal outcomes, partly driven by rapid emergence of resistance, particularly via AmpC overexpression (Ding et al., 2023).

Therefore, clinical evidence supporting aztreonam-avibactam in MBL-producing *P. aeruginosa* remains limited (Pina-Sánchez et al., 2025; Mularoni et al., 2021; Sempere et al., 2022). Where adopted, synergy testing is strongly recommended, although it is not routinely available in most clinical microbiology laboratories. A small study demonstrated a limited synergistic effect (14.3%) in CRPA, using the gradient strip stacking method (Blanco et al., 2025). Therefore, when cefiderocol is unavailable, contraindicated, or inactive *in vitro*, aztreonam-based regimens may be considered as an alternative treatment option, provided that susceptibility is confirmed by synergy testing.

### 5.4 Role of older antimicrobial agents

When newer antimicrobial agents are unavailable, older antibiotics may still provide useful therapeutic alternatives for CRPA. Aminoglycosides, fosfomycin and colistin may retain activity against a proportion of CRPA, although their overall role is limited by toxicity and pharmacokinetic constraints.

Historically, colistin-based regimens were the cornerstone of treatment for infections caused by MBL-producing *P. aeruginosa* before the introduction of newer anti-pseudomonal agents. However, current international guidelines advise against routine use of polymyxins due to concerns regarding nephrotoxicity and inferior clinical outcomes. Aminoglycosides may retain activity against selected MBL-producing *P. aeruginosa* and can be considered in urinary tract infections. Intravenous fosfomycin has also been proposed as a companion agent in combination strategies, although supporting evidence remains limited and largely derived from observational studies. Overall, the clinical utility of these agents is constrained by significant toxicity, suboptimal tissue penetration, and unpredictable pharmacokinetic profiles, which collectively limit their use in critically ill patients (Pina-Sánchez et al., 2025; Hidalgo-Tenorio et al., 2024).

### 5.5 Emerging therapeutic strategies

Several novel antimicrobial agents are currently under investigation to address the growing challenge posed by multidrug-resistant Gram-negative pathogens, with particular attention to *P. aeruginosa*, for which therapeutic options remain limited. However, the level of evidence supporting these emerging agents varies considerably. For most compounds, activity against MBL is currently supported primarily by *in vitro* susceptibility studies, whereas clinical evidence remains limited.

Cefepime/taniborbactam combines cefepime with a novel bicyclic boronate β-lactamase inhibitor and represents one of the first agents with direct inhibitory activity against MBL enzymes, while also inhibiting β-lactamases belonging to the other Ambler classes. In contrast to agents such as cefiderocol, which retain activity through stability to MBL-mediated hydrolysis, taniborbactam exerts direct enzymatic inhibition (Van Helden et al., 2025). *In vitro* studies have demonstrated substantial activity against CRPA, including isolates harboring multiple resistance mechanisms. A multicenter Spanish study reported activity rates of 63%−67% against MBL-producing *P. aeruginosa* strains, exceeding those of comparator agents (Hernández-García et al., 2022). However, activity is not uniform across MBL variants, with reduced efficacy reported against certain IMP-like, SIM-1, NDM-9 and VIM-83 producers (Le Terrier et al., 2024). Nevertheless, these promising *in vitro* findings still require confirmation in clinical studies. A microbiological analysis of the phase 3 CERTAIN-1 trial demonstrated favorable outcomes of cefepime/taniborbactam against resistant pathogens; however, the number of *P. aeruginosa* isolates, especially MBL producers, was too small to allow pathogen-specific conclusions (Moeck et al., 2024).

Other agents in development, including meropenem/xeruborbactam and cefepime/zidebactam, have also demonstrated encouraging *in vitro* activity. Xeruborbactam is a cyclic boronate β-lactamase inhibitor with broad activity against Gram-negative pathogens producing serine β-lactamases or MBL, including partial inhibitory activity against certain IMP-like enzymes, not effectively targeted by taniborbactam (Le Terrier et al., 2024). When combined with meropenem, it has demonstrated promising *in vitro* activity against MBL-producing bacteria, especially *Enterobacterales* (Lomovskaya et al., 2023; Nelson et al., 2020), although activity against *P. aeruginosa* is more variable, likely due to efflux-mediated reduced intracellular accumulation and reduced activity against specific IMP variants (e.g., IMP-10) (Le Terrier et al., 2024). Similarly, the combination of cefepime/zidebactam represents a promising option against MDR Gram-negative pathogens. Zidebactam acts as a β-lactam enhancer by binding to PBP2, thereby potentiating the activity of cefepime even against MBLs. In a collection of clinical isolates from the UK National Reference Laboratory, *in vitro* cefepime/zidebactam inhibited approximately 95% of *P. aeruginosa* isolates carrying ESBLs or MBLs, whereas comparator agents showed susceptibility rates below 15% (Mushtaq et al., 2021).

Murepavadin, a first-in-class outer membrane protein-targeting antibiotic with selective activity against *P. aeruginosa*, represents an innovative non-β-lactam approach. It targets lipopolysaccharide transport protein D (LptD), disrupting outer membrane biogenesis. Its narrow spectrum is expected to minimize microbiota disruption and selective pressure on non-target organisms, while maintaining potent activity against *P. aeruginosa* (Martin-Loeches et al., 2018). *In vitro* surveillance studies reported inhibition rates of 95%-99% of the isolates (Sader et al., 2018a,b). Nevertheless, clinical development was subsequently discontinued because of concerns about nephrotoxicity observed during clinical trials in patients with ventilator-associated pneumonia.

The combination of cefiderocol with taniborbactam has also been explored as a strategy to enhance activity against strains with additional resistance mechanisms. Although cefiderocol is intrinsically stable to MBL hydrolysis, reduced susceptibility has been increasingly observed when multiple resistance determinants coexist. Preliminary *in vitro* data suggest activity against approximately 95% of NDM-producing *P. aeruginosa* isolates, supporting a promising role notably in settings with high NDM-prevalence (Kocer et al., 2025).

Finally, bacteriophages, which were first developed more than a century ago, have recently regained considerable interest as a potential treatment option for CRPA. Although clinical evidence remains very limited and largely restricted to case reports and small observational studies, phage therapy represents a promising adjunctive strategy, with potential use in combination with conventional antibiotics to enhance bacterial eradication through synergistic interaction and potentially overcome antimicrobial resistance. In addition, inhaled phage therapy is an attractive approach for chronic respiratory infections, notably in patients with cystic fibrosis, where high local phage concentrations can be achieved while minimizing systemic exposure (Horcajada et al., 2019; Chan et al., 2025; Terlizzi et al., 2026).

### 5.6 Proposed treatment algorithm for MBL-producing *P. aeruginosa* infections

MBL-producing *P. aeruginosa* represents one of the most challenging antimicrobial resistance phenotypes encountered in clinical practice. The mechanisms of action of currently available and emerging antimicrobial agents are summarized in Figure 1. Siderophore cephalosporins such as cefiderocol exploit iron uptake pathways and may retain activity against isolates with several β-lactam resistance mechanisms. In the setting of MBL-producing *P. aeruginosa*, combinations such as aztreonam with avibactam are of particular interest because aztreonam remains stable to MBL hydrolysis, while avibactam inhibits co-produced serine β-lactamases. Conversely, emerging β-lactamase inhibitors, including taniborbactam and xeruborbactam, are being investigated for their potential activity against selected serine lactamases and MBL. Additional approaches targeting virulence-associated pathways or bacterial survival mechanisms, such as murepavadin and bacteriophage-based therapies, represent promising strategies currently under investigation (Figure 1).

**Figure 5:**
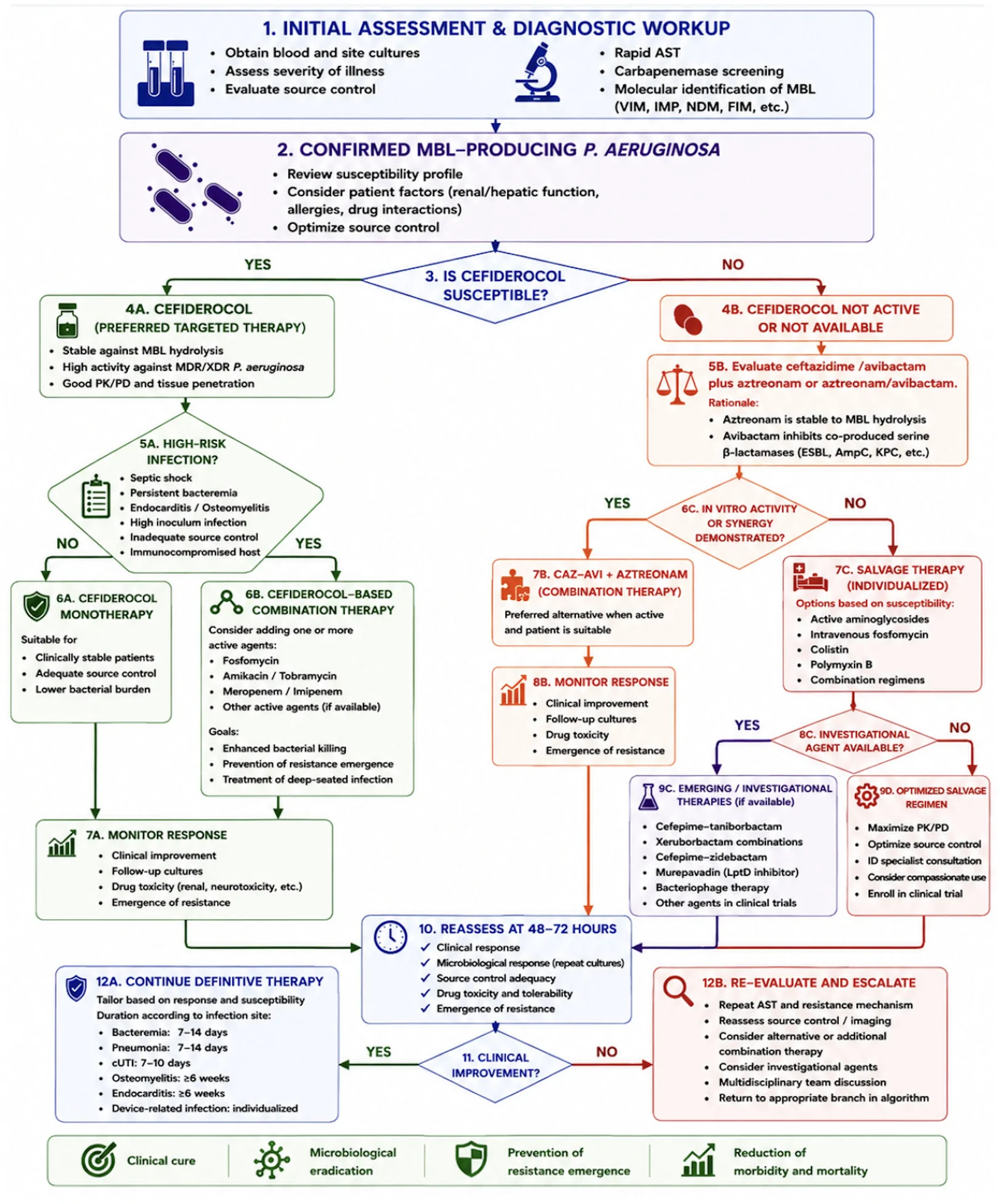
Proposed therapeutic algorithm for infections caused by metallo-β-lactamase-producing *Pseudomonas aeruginosa*. AmpC, class C β-lactamase; AST, antimicrobial susceptibility testing; CAZ-AVI, ceftazidime-avibactam; ESBL, extended-spectrum β-lactamase; FIM, florence imipenemase; ID: infectious diseases; IMP, imipenemase; KPC, *Klebsiella pneumoniae* carbapenemase; MBL, metallo-β-lactamase; MDR, multidrug-resistant; MIC, minimum inhibitory concentration; NDM, New Delhi metallo-β-lactamase; PK/PD, pharmacokinetics/pharmacodynamics; UTI, urinary tract infection; VIM, Verona integron-encoded metallo-β-lactamase; XDR: extensively drug-resistant. The algorithm integrates current evidence on cefiderocol, ceftazidime-avibactam in combination with aztreonam, salvage therapies and emerging or investigational treatment strategies non-yet standard of care. Treatment should be guided by infection severity, adequacy of source control, antimicrobial susceptibility testing and confirmation of the underlying resistance mechanism. Cefiderocol is positioned as the preferred option when susceptibility is demonstrated, while ceftazidime-avibactam plus aztreonam may be considered when *in vitro* activity or synergy is demonstrated. Combination therapy should be reserved for selected cases, including severe infections, high bacterial burden, persistent bacteremia, or suboptimal source control. The algorithm is based on contemporary guidelines and recent evidence on difficult-to-treat resistant *P. aeruginosa* infections. Figure design was assisted by ChatGPT (OpenAI).

Based on current guidelines and available evidence, a proposed treatment algorithm for infections caused by MBL-producing *P. aeruginosa* is shown in Figure 5.

## 6 Conclusions

The global epidemiology of MBL-producing *P. aeruginosa* is characterized by marked geographic heterogeneity, continuous molecular evolution, and the dissemination of high-risk clones, underscoring the importance of integrating local epidemiology into therapeutic decision-making. Accurate management requires a mechanism-based approach that combines rapid diagnostics, phenotypic susceptibility testing, source control, optimized antimicrobial exposure, and antimicrobial stewardship. Although cefiderocol currently represents the preferred therapeutic option for MBL-producing *P. aeruginosa*, emerging agents and novel strategies may further expand future treatment options.

By integrating molecular microbiology, clinical infectious diseases and antimicrobial stewardship into a mechanism-based therapeutic framework, this review provides a practical approach to individualized management while highlighting key priorities for future research.

## Footnotes

1 Available at https://www.ecdc.europa.eu/en/publications-data/surveillance-antimicrobial-resistance-europe-2024-data.

2 Available at https://www.ecdc.europa.eu/en/publications-data/antimicrobial-resistance-eueea-ears-net-annual-epidemiological-report-2024.

3 Available at https://www.epicentro.iss.it/antibiotico-resistenza/ar-iss-rapporto-pseudomonas-aeruginosa.

4 EUCAST guidelines for detection of resistance mechanisms (2017); available at: https://www.eucast.org/bacteria/important-additional-information/resistance-detection/.

5 CLSI M100 Performance Standards for Antimicrobial Susceptibility Testing (2026); available at: t https://clsi.org/shop/standards/m100/.
